# The role of lipid metabolism and creeping fat in disease assessment and prediction for Crohn’s disease

**DOI:** 10.3389/fimmu.2026.1821405

**Published:** 2026-05-25

**Authors:** Ying Lei, Runjie Shi, Zihong Cai, Yiqing Li, Xiaowei Qiu, Xiang Li, Shanping Wang, Yan Sun

**Affiliations:** Department of Gastroenterology, Guangdong Provincial Key Laboratory of Major Obstetric Diseases, Guangdong Provincial Clinical Research Center for Obstetrics and Gynecology, The Third Affiliated Hospital, Guangzhou Medical University, Guangzhou, Guangdong, China

**Keywords:** creeping fat, Crohn’s disease, disease assessment, lipid metabolism, review

## Abstract

Crohn’s Disease (CD) is a chronic disease that often leads to disability. Effective monitoring of the disease is essential for delaying progression and improving outcomes. Traditional assessment methods have some limitations, such as invasiveness, delayed response, or inability to evaluate transmural lesions. This review focuses on lipid metabolism and creeping fat (CF) and explores their roles in disease prediction and assessment of CD. Through literature review, this study summarized the currently commonly used methods for assessing the condition, and also summarized the relationships among blood lipids, inflammation, and adipose tissue, as well as their effects during the disease activity period of CD. The methods for CD to assess the condition include endoscopy, radiological examination and endoscopic ultrasound and so on. During active phase, patients often show characteristic dyslipidemia. These lipid changes are closely related to systemic inflammation severity. As a unique feature in CD, CF consists of mesenteric fat expansion and bowel wrapping, contributing to intestinal inflammation, fibrosis, and stricture formation. Immune responses, microbial factors, and molecular pathways are involved in this process. Current imaging techniques enable the assessment of CF for predicting surgical risks and postoperative recurrence. Considering the close correlation between lipid levels and CF and the inflammation during the active phase of CD, there is potential for disease assessment. In the future, we should focus on the application of lipid levels and CF in assessing and predicting the activity of CD.

## 1 Introduction

Crohn’s disease (CD) is one of the main types of inflammatory bowel disease. It is a chronic, non-specific, recurrent inflammatory disease of unknown cause. Disease monitoring is important for evaluating disease activity, predicting prognosis, and guiding treatment decisions (1, 2). In the 20th century, CD was considered as a typical Western illness (3). However, the number of CD patients has increased worldwide. In newly industrialized regions such as Asia, although the incidence and prevalence of CD remains lower than in Western countries, the number of new cases is increasing rapidly, making it a significant public health concern (4). The etiology and mechanism of CD remain unclear. Epidemiological studies suggest that this may be a complex interactive outcome mediated by a combination of environmental factors, lifestyle factors, genetic susceptibility, alterations in the gut microbiota, and abnormal immune responses (3, 5–7).

CD is a chronic granulomatous disease that can affect any part of the gastrointestinal tract. The disease is characterized by transmural inflammation. Typical clinical manifestations include abdominal pain, diarrhea, anorexia, weight loss, and fatigue (8). At the same time, it can progress to complications such as intestinal stenosis, fistulas and abscesses, which are the main reasons for patients’ repeated visits, hospitalizations and surgeries (9). The clinical manifestations of CD are not limited to the gastrointestinal tract. Some patients may also experience extraintestinal symptoms. Extraintestinal manifestations (EIMs) often affect joints, skin and eyes, and can also affect important organs such as the liver, lungs and pancreas, further increasing the burden of the disease (10).

As a disease with a high potential for disability, CD is associated with a poor long-term prognosis. Long-term follow-up studies show a heavy disease burden. The cumulative incidence rate of disabling diseases is as high as 74.6% (11, 12). CD adversely impacts both physical and mental health, often impairing social functioning and increasing overall disease burden (13). Faced with this high risk of disability and the heavy burden on quality of life, preventing disease progression is a major goal of disease management.

In recent years, the role of lipid metabolism disorders and creeping fat (CF) in CD has gradually attracted attention. CF is one of the characteristic pathological changes in CD, where it abnormally proliferates along the intestinal wall and wraps around the inflamed intestinal segment. Through complex immune regulation and interaction with intestinal microorganisms, it participates in the progression of inflammation and the process of intestinal fibrosis (14). At the same time, dyslipidemia is closely related to systemic inflammatory levels, which may reflect the disease activity status and affect prognosis (15). Therefore, systematically exploring the role of lipid metabolism and CF in CD can help improve the accuracy of disease assessment and provide new potential targets for prognosis prediction and individualized treatment.

## 2 Literature search strategy

To clarify the roles of lipids and CF in CD, we conducted a comprehensive search of the PubMed database up to October 2025. Search terms included combinations of the following keywords: “Inflammatory Bowel Disease”, “Crohn’s Disease”, “Lipid”, “Cholesterol”, “Dyslipidemia”, “Lipoprotein”, “Triglyceride”, “Creeping fat”, “Crawling fat” and “Mesenteric fat”. Exclusion criteria included non-English literature, irrelevant to the topic, specific population restrictions, etc. Key information relevant to this study was extracted from the selected literature.

## 3 Methods for the assessment of CD activity

Clinically, several tools are commonly used to assess CD. The Simple Endoscopic Score for Crohn’s Disease (SES-CD) is one of these tools based on endoscopic findings. The Crohn’s Disease Activity Index is another commonly used tool based on clinical symptoms. Clinical symptoms do not always reflect intestinal inflammation in CD because mucosal inflammation and tissue injury can progress without clear symptoms. Symptoms often appear later than objective inflammatory changes, so relying solely on the symptoms reported by patients may delay the necessary intervention measures. A comprehensive assessment combining objective methods such as endoscopy, imaging, and biomarkers is very important (16).

### 3.1 Endoscopy

Endoscopic assessment is typically conducted at three key moments, including during the initial diagnosis, early after the start of treatment, and during long-term follow-up. Active inflammation can influence treatment decisions, so repeated endoscopy may be needed to assess active inflammation. Repeated endoscopy may also be required after major treatment changes (17). Endoscopy remains a main method for assessing CD activity, but it is an invasive procedure with several limitations. Many patients have low tolerance for repeated endoscopic examinations. It may carry procedure-related complications. Intestinal strictures, adhesions, or severe inflammation can increase procedural difficulty. Endoscopy may miss lesions in the proximal small bowel, leading to false-negative results. In addition, endoscopy cannot assess extraintestinal manifestations (18–20). Capsule endoscopy is a minimally invasive technique. It allows non-invasive visualization of the small bowel and colonic mucosa. Compared with traditional examination methods, CE has a higher detection capability in identifying proximal and mild small intestinal lesions. CE also has several limitations, including the inability to conduct tissue sampling, as well as potential procedural risks such as capsule retention and intestinal perforation. Adequate bowel preparation is required before the procedure. It is contraindicated in specific patient populations. Additionally, the examination process is time-consuming. Due to issues with intestinal transit, some patients may experience incomplete visualization. CE is limited to the assessment of the mucosal surface. It cannot evaluate deep intestinal wall changes such as the extent of transmural inflammation, strictures, or penetrating disease. Extraintestinal complications are also not evaluated by this method (18, 21).

### 3.2 Radiological examination

Radiological examinations are important for diagnosing CD. They are also used in disease progression monitoring and management. They complement endoscopic assessment, especially for evaluating small bowel lesions and intestinal strictures. CT enterography (CTE) and magnetic resonance enterography (MRE) are cross-sectional imaging techniques optimized for small bowel imaging (22). CTE has high spatial resolution, allowing clear visualization of transmural inflammation and extraintestinal complications. It effectively identifies lesions in the proximal and mid-ileum. These areas are often not accessible by coloscope (23). As a non-invasive imaging method, MRE is becoming more and more important in the diagnosis and follow-up of CD. It allows for the assessment of the extent of transmural lesions and extraintestinal complications without exposing patients to radiation risks. Transmural healing is now considered an important treatment goal in CD. MRE can evaluate deep structures of the intestinal wall and assess inflammatory activity. Because of these advantages, international guidelines recommend MRE as a first-line imaging method (20). However, MRE has limitations compared to CTE, including higher cost, less availability, greater variability in image quality, and lower spatial resolution (22). Research shows that CTE performs well in assessing disease activity, and its performance is comparable to that of MRE (18).

### 3.3 Intestinal ultrasound

Intestinal ultrasound (IUS) is a non-invasive, cost-effective, reproducible, and radiation-free imaging method. It plays an important role in the diagnosis and follow-up of CD. IUS can accurately assess bowel wall thickness, stratification, vascularity, and mesenteric status. It can also effectively detect complications such as strictures, fistulas, and abscesses. IUS findings are closely correlated with clinical symptoms, endoscopic findings, and laboratory markers such as fecal calprotectin. This makes it an effective tool for assessing disease activity, predicting treatment response, and estimating the risk of recurrence. IUS has a certain degree of operator dependence, and it can be affected by bowel gas and obesity (24–27).

### 3.4 Endoscopic ultrasonography

Endoscopic ultrasonography combines endoscopic and ultrasound techniques. The probe can be placed in direct contact with the intestinal wall. This allows a detailed observation of the superficial and deep structures of the gastrointestinal tract, such as the layers of the intestinal wall and the lymph nodes around the intestine. EUS has unique value in disease diagnosis, activity assessment, severity grading and prognosis evaluation, and it can provide supplementary information that is usually difficult to obtain through other imaging methods. However, EUS is a technically complex procedure requiring extensive training to perform correctly. The interpretation of the results is highly dependent on the operator. These limitations restrict the wider use of EUS in CD assessment (25, 28).

### 3.5 Biomarkers

Biomarkers hold significant value in the assessment of disease activity and prognostic prediction in CD. C-reactive protein (CRP) and fecal calprotectin (FCP) are currently the most widely used biomarkers in clinical practice. Elevated CRP levels reflect systemic inflammation. Increased CRP is associated with disease progression and an increased risk of hospitalization. FCP is more specific for intestinal inflammation. It performs better than CRP in evaluating endoscopic activity and recurrence. However, FCP levels vary widely between individuals. Sensitivity is lower in isolated ileal disease and proctitis. Fecal immunochemical tests (FIT) have shown potential in several studies. And other emerging biomarkers, such as oncostatin M, leucine-rich alpha-2-glycoprotein, fecal myeloperoxidase, and fecal microRNAs, have also shown potential in research settings. But the levels of biomarkers may be affected by various factors, leading to risks of misdiagnosis or missed diagnosis. Unnecessary examinations may be performed, and some patients may experience increased anxiety. There may be delays in disease management (29, 30). Thus biomarkers are mainly used as supportive tools for disease monitoring. Results need to be interpreted together with other clinical findings.

In summary, there are multiple methods to assess CD, including endoscopy, imaging, and biomarkers. Disease management should be individualized and dynamic. The assessment results should be interpreted in conjunction with clinical manifestations to optimize treatment decisions and improve long-term prognosis.

## 4 Research progress on blood lipids in CD

### 4.1 Blood lipids and inflammation

Blood lipids include cholesterol, neutral fats, phospholipids, and free fatty acids. Clinically, commonly used indicators include TC, TG, High-density lipoprotein cholesterol (HDL-C), and Low-density lipoprotein cholesterol (LDL-C) (31). More and more evidence indicates a close bidirectional association between the inflammatory response and lipid metabolism. Dyslipidemia can increase inflammatory mediator levels, such as tumor necrosis factor-alpha (TNF-α) and interleukin-6 (IL-6) (32). Cholesterol is a major component of the plasma membrane. It helps maintain membrane integrity, and also affects cellular function by regulating cell signaling pathways (33, 34). Increased serum cholesterol levels can change Kv channel turnover and recruitment in myocytes. This may affect cellular homeostasis (34). The cholesterol transporters ATP-Binding Cassette A1 (ABCA1) and G1 (ABCG1) mediate the efflux of active cholesterol to apolipoprotein A-I and low-density lipoprotein (LDL), respectively. Studies have indicated that cholesterol accumulation in mice deficient in ABCA1 and ABCG1 leads to the hyperactivation and proliferation of monocytes and neutrophils, accompanied by upregulated expression of inflammatory genes (35, 36). The accumulation of cholesterol can lead to increased activation and proliferation of monocytes and neutrophils. The process is accompanied by the upregulation of inflammatory gene expression. The accumulation of cholesterol within mitochondria can damage mitochondrial function. It also disrupts the normal assembly of respiratory supercomplexes. This functional impairment ultimately triggers oxidative stress, leading to tissue injury. LDL-C particles can activate platelets and damage the vascular endothelium. These effects initiate and sustain cellular inflammatory processes. Smaller LDL-C particles showed greater endothelial permeability. After oxidation occurs, these particles may cause more severe cell damage (32). Hsu et al. found a positive correlation between levels of small dense low-density lipoprotein cholesterol (sdLDL-C) and systemic inflammatory markers. These markers include high-sensitivity C-reactive protein (hs-CRP) and leukocyte counts characterized predominantly by neutrophilia (37). Krishnamurthy et al. reported that Apolipoprotein E (APOE) polymorphisms significantly influence the levels of multiple lipid markers, including LDL, HDL, small dense LDL (sdLDL), and oxidized LDL (oxLDL). Specifically, the APOE4 allele is associated with higher sensitivity to oxidative stress and inflammation induced by oxLDL. It is also linked to elevated levels of inflammatory markers such as hs-CRP and myeloperoxidase (MPO) (38). High-density lipoprotein (HDL) possesses anti-proliferative, anti-inflammatory, and anti-oxidative properties. These functions include binding lipopolysaccharides and removing oxidized lipids from low-density lipoproteins (LDL) (36).

**Figure 1:**
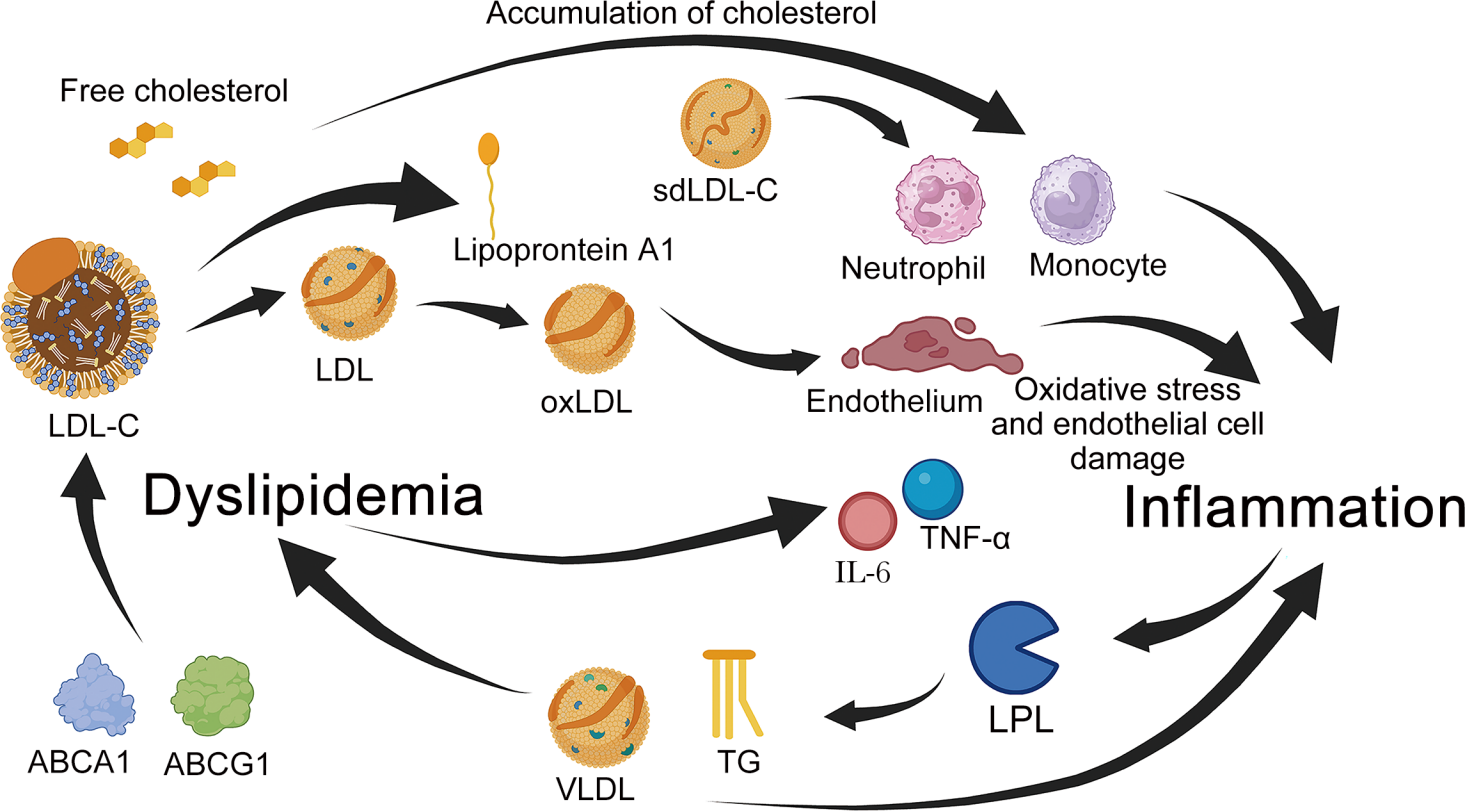
The bidirectional interaction between dyslipidemia and inflammation. Dyslipidemia can increase TNF-α and IL-6. The cholesterol transporters ABCA1 and ABCG1 mediate the efflux of active cholesterol to apolipoprotein A-I and LDL, respectively. Cholesterol accumulation in deficient in ABCA1 and ABCG1 leads to the hyperactivation and proliferation of monocytes and neutrophils. SdLDL-C contributes to the increase of neutrophils. LDL-C particles show great endothelial permeability. After oxidation occurs, these particles may cause more severe cell damage, leading to inflammation. Inflammation can affect the degradation of TGs by inhibiting the activity or synthesis of LPL and hepatic lipase, leading to elevated levels of TG and VLDL. Created with BioRender.com. ABCA1, ATP-Binding Cassette A1; ABCG1, ATP-Binding Cassette G1; LDL, low-density lipoprotein; SdLDL-C, small dense lipoprotein cholesterol; LDL-C, low-density lipoprotein cholesterol; TG, triglyceride; LPL, lipoprotein lipase; VLDL, very low-density lipoprotein.

A prolonged or chronic inflammatory state plays an important role in persistent dyslipidemia. In metabolic syndrome, this chronic inflammation is considered as a core component. It is closely associated with pro-inflammatory cytokines such as IL-6 and TNF-α (39). Research by Poznyak et al. showed that inhibiting TNF-α can regulate lipid levels by weakening the inflammatory response (40). TGs and its primary carrier, very low-density lipoprotein (VLDL), are intimately linked to inflammatory processes. Inflammation can affect the degradation of TGs by inhibiting the activity or synthesis of lipoprotein lipase (LPL) and hepatic lipase, leading to elevated levels of TG and VLDL. Conversely, TGs and VLDL themselves can also exacerbate the inflammatory response. This bidirectional mechanism is particularly common in autoimmune diseases (41). In summary, a close and mutually reinforcing association exists between lipids and inflammation. This relationship can form a vicious cycle (**Figure 1**).

### 4.2 Changes in blood lipids during the active phase of CD

Persistent lipid metabolism abnormalities are closely linked to disease activity in CD (15). A large number of studies have shown that there is a significant correlation between the serum levels of TC, TG, LDL-C and HDL-C (42).Among these lipid markers, HDL-C is often considered protective because higher HDL-C levels are linked to a lower risk of CD (43). During the active phase of the disease, patients with CD often show a characteristic pattern of dyslipidemia. Many studies have reported a reduction in serum TC, HDL-C, and LDL-C levels during acute flares or active disease. This reduction in lipid components is a characteristic feature of metabolic abnormalities during the acute phase of CD (44, 45). This phenomenon was further corroborated by the study of Wang et al., which found that CD activity was negatively correlated with TC, HDL-C, and LDL-C levels, while showing no significant association with TG (46).

However, several studies have revealed slightly different lipid profiles. For example, Koutroumpakis et al. reported a dyslipidemia pattern, which was characterized by a decrease in TC and LDL-C, while TG and HDL-C increased. Importantly, the severity of lipid metabolism abnormalities is linked to disease severity. The extent of this lipid metabolism disorder is positively correlated with the severity of the clinical disease, such as the surgery rates and the frequency of hospitalizations. The underlying mechanism is complex. It may involve malabsorption caused by ileal lesions and changes in hepatic lipoprotein metabolism. These metabolic changes are driven by systemic inflammation (15). Among various lipid markers, HDL-C shows unique predictive value. Research shows that low levels of HDL-C are associated with more severe disease activity and a poor long-term prognosis in CD. More importantly, HDL-C levels change more slowly over time. Compared with markers such as CRP, HDL-C is less affected by short-term fluctuations, making HDL-C more stable for long-term assessment. This feature makes low HDL-C a promising, more persistent, and stable clinical biomarker for assessing CD severity and predicting long-term prognosis (47). Iwatani et al. reported differences in lipid profiles between patients with CD and healthy controls. These lipid changes are closely related to disease activity. These lipid levels were positively correlated with CDAI and erythrocyte sedimentation rate (ESR), while negatively correlated with nutritional indicators such as hemoglobin and albumin (48).

The application of biologic agents helps clarify the link between blood lipids and inflammation in CD. Koutroubakis et al. found that in patients treated with infliximab who achieved clinical remission, levels of TC and HDL-C increased simultaneously as systemic inflammation lessened and nutritional status improved (49). A similar pattern was reported by Sandborn et al. in a study on tofacitinib. While effectively alleviating the inflammation of CD, there was also an increase in the levels of TC, HDL-C and LDL-C. These changes in blood lipid levels are reversible after the treatment is stopped. Importantly, the increase in lipid levels showed a significant negative correlation with the decrease in hs-CRP. This indicates that the rise in lipid levels is not an isolated phenomenon but rather a predictable and reversible concomitant biomarker during the improvement of CD inflammation (50). These studies confirm a close link between lipid abnormalities and acute inflammation in CD. It is suggested that blood lipid levels may serve as a biomarker for monitoring the activity of the disease.

### 4.3 Mechanisms of lipid changes during the active phase of CD

Dyslipidemia is not merely a simple accompanying phenomenon in the development of CD. The primary function of cholesteryl ester transfer protein (CETP) is to mediate the transfer of cholesteryl esters from HDL to LDL and VLDL. Tao et al. have shown that inhibiting CETP can reduce the risk of developing CD, and this protective effect is mediated by HDL-C (43). In an inflammatory environment, lipid metabolism is closely linked to immune activation. LPL is an enzyme located on the surface of the vascular endothelium responsible for hydrolyzing circulating TGs. Pro-inflammatory cytokines such as TNF-α, IL-1, and IFN-γ can inhibit lipoprotein lipase activity. As a result, TGs hydrolysis is reduced. This change leads to an abnormal lipoprotein profile. Research shows that LPL activity is significantly influenced by factors such as serum CRP levels, disease duration, and the ileocolonic phenotype. These factors collectively participate in the pathological process of dyslipidemia in CD patients by influencing LPL activity (51). In addition, levels of anti-inflammatory HDL and its major component apolipoprotein A-I (apoA-I) are reduced in CD. This reduction weakens the ability to remove free radicals, inhibit monocyte activation, and reduce TNF-α production. These findings suggest that metabolic abnormalities in CD are not passive consequences. Instead, they actively promote the persistence of inflammation by weakening anti-inflammatory pathways. As a result, a vicious cycle may develop (52).

**Figure 2:**
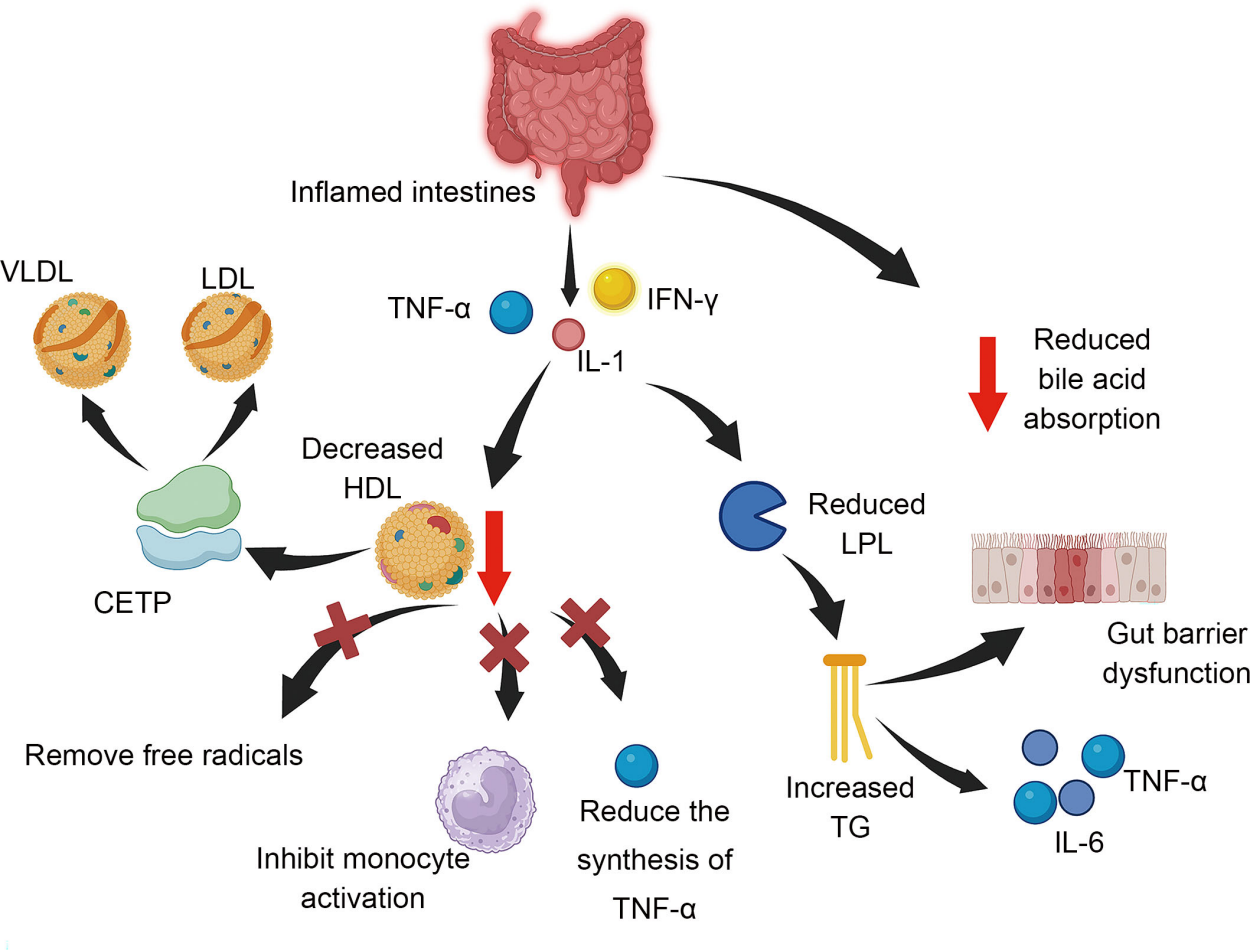
The mechanism of lipid changes during CD activity period. CETP promotes the transfer of cholesterol esters from HDL to LDL and VLDL. A decrease in HDL reduces the ability to eliminate free radicals, inhibit monocyte activation, and reduce the production of TNF-α. LPL is responsible for hydrolyzing triglycerides in the circulation. TNF-α, IL-1 and IFN-γ can inhibit the activity of lipoprotein lipase, resulting in a reduction in the hydrolysis of TGs. An increase in TGs is accompanied by elevated levels of pro-inflammatory cytokines such as TNF-α and IL-6, exacerbating chronic intestinal inflammation. Chronic inflammation of the intestines can lead to impaired bile acid absorption. Created with BioRender.com. CD, Crohn’s disease; CETP, cholesteryl ester transfer protein; HDL, high-density lipoprotein; LDL, low-density lipoprotein; VLDL, very low-density lipoprotein; TNF-α, tumor necrosis factor-α; LPL, lipoprotein lipase; TG, triglyceride.

It has been reported that the expression of the fatty acid transporter CD36 is abnormal in the small intestine of patients with CD, with a significantly reduced number of positive cells observed in the damaged colonic mucosa. At the same time, bile acid malabsorption also affects the function of CD4+ effector T cells (Teff) in the ileum. The high-level expression of Teff cytokines in tissues is a hallmark of CD. Bile acid malabsorption is common among patients with CD and may lead to steatorrhea and hypocholesterolemia (53). The triglyceride-glucose (TyG) index is a novel biomarker calculated from fasting triglyceride and glucose levels. In recent years, due to its simplicity and reliability, the TyG index has been confirmed to be closely associated with the risk of various chronic diseases. The clinical value of the TyG index has been further validated in studies on CD. Research indicates that a higher TyG index is significantly associated with an increased risk of adverse clinical outcomes in patients with CD, including intestinal obstruction, fistula formation, and the need for surgical intervention. The underlying pathophysiological mechanisms involve multiple pathways. The state of metabolic dysregulation represented by a high TyG index is accompanied by elevated levels of pro-inflammatory cytokines such as TNF-α and IL-6, which persistently exacerbate chronic intestinal inflammation. In addition, the accompanying hyperlipidemia will increase the level of oxidative stress, thereby damaging the intestinal epithelial cells and disrupting the integrity of the intestinal barrier. Ultimately, chronic inflammation, excessive oxidative stress, and accelerated fibrosis all work together to drive the progression of CD and the occurrence of complications (54). This suggests that the use of lipid metabolism indicators may provide a critical window for reflecting the interaction between local intestinal lesions and systemic inflammation in patients with CD (**Figure 2**).

## 5 Research progress on CF in CD

### 5.1 Definition and characteristics of CF

CF is a common pathological feature of CD. It is also known as mesenteric adipose tissue hypertrophy or fat encapsulation. This feature is distinctive in CD but rarely observed in patients with ulcerative colitis (UC) (14, 55). CF refers to a pathological phenomenon where mesenteric adipose tissue (MAT) abnormally proliferates at the site of intestinal inflammation and extends to envelop the intestinal loops. It is predominantly observed in the small intestine, particularly the ileum. According to the traditional diagnostic criteria, the range of intestinal mesentery fat infiltration must cover more than 50% of the intestinal circumference to be diagnosed as CF. However, recent observations suggest that this definition may be too strict. Milder degrees of fat creeping are frequently observed in surgical specimens, indicating that the actual prevalence of CF in CD may be underestimated (56, 57). The areas affected by CF often show obvious intestinal fibrosis, and this fibrosis may even extend into the muscular layer. As a result, it is commonly observed in patients with fibrotic complications, such as intestinal strictures (58). Many studies have confirmed that the presence of CF is closely associated with intestinal fibrosis, luminal stenosis, the occurrence of bowel obstruction, and the risk of postoperative recurrence during the course of CD (56, 59). An MRI study indicated that the prevalence of CF in a CD cohort was 21.1% (60).

### 5.2 Mechanisms of CF formation

The formation of CF involves a multitude of cellular and molecular mechanisms. Adipose tissue is composed of mature adipocytes, preadipocytes, endothelial cells, and other cell types. These cells play a critical role in the inflammatory process of CD by secreting cytokines and through intercellular interactions (55, 61). Research indicates that impaired intestinal barrier function can cause the translocation of microorganisms such as the gut bacterium *C. innocuum* and their metabolites to the peritestinal area. This translocation regulates the local immune microenvironment and drives the formation of CF (58). Wu et al. discovered that the upregulation of indoleamine 2,3-dioxygenase 1 (IDO1) in macrophages enhances kynurenine metabolism, resulting in the abnormal enrichment of L-kynurenine in CF. This process further promotes the pathological expansion of MAT through adipogenesis mediated by the aryl hydrocarbon receptor (62).

**Figure 3:**
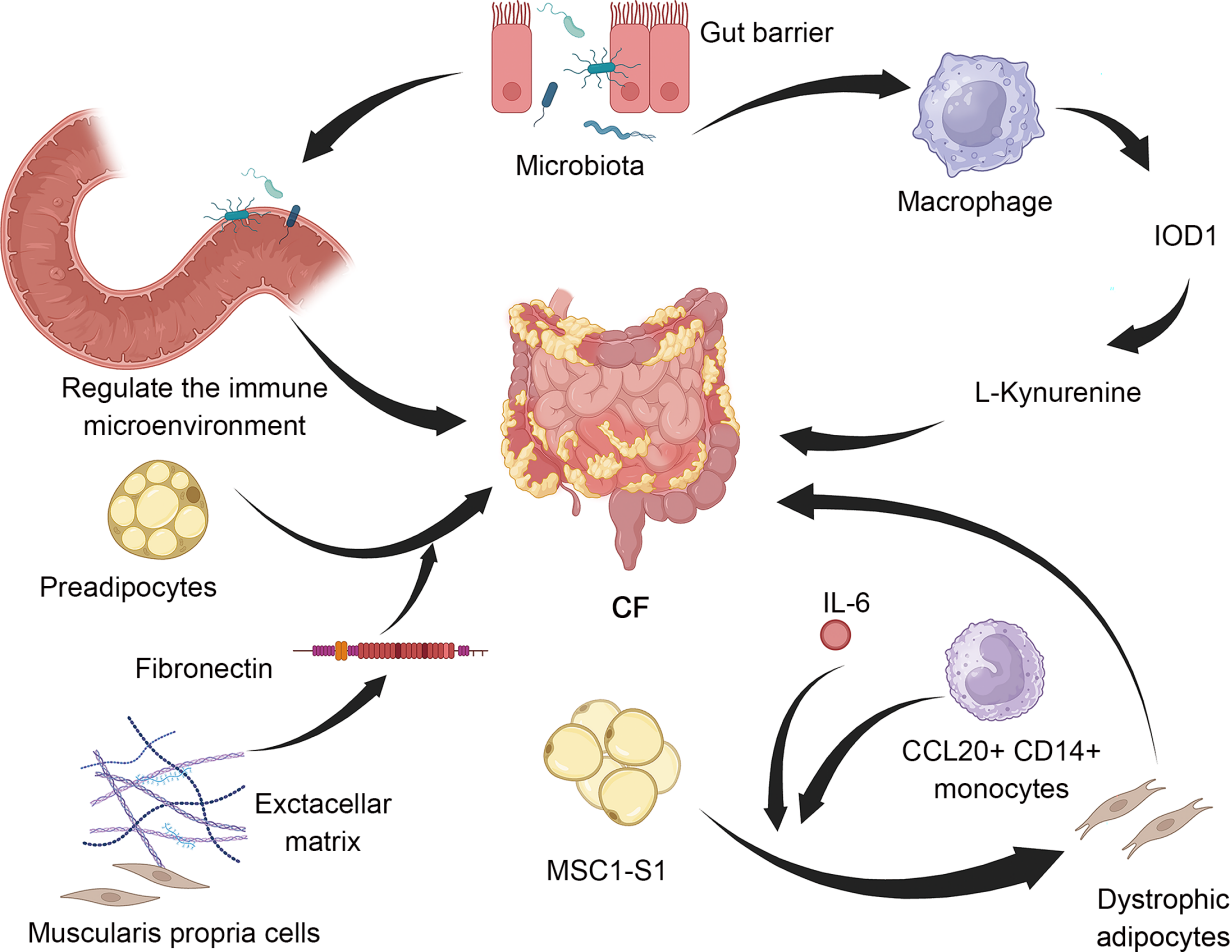
The mechanism of CF formation. Impaired intestinal barrier can cause the migration of microorganisms and their metabolites from the intestinal lumen to the peritestinal area, regulating the local immune microenvironment and promoting the formation of CF. The upregulation of IDO1 in macrophages leads to abnormal accumulation of L-kynurenine in the CF, promoting the pathological expansion of MAT. The muscle layer cells produce a unique ECM scaffold, which maintains a direct connection with the CF covering them. Within this matrix, fibronectin is a key factor driving the migration of preadipocytes out of the mesenteric adipose tissue, contributing to CF formation. A subpopulation of MSC1-S1 is significantly expanded within CF. Under stimulation by CCL20+CD14+ monocytes and IL-6, this subpopulation accelerates differentiation into dystrophic adipocytes, driving the aberrant formation of CF. Created with BioRender.com. CF, creeping fat; IDO1, indoleamine 2,3-dioxygenase 1; MAT, mesenteric adipose tissue; ECM, extracellular matrix; MSC1-S1, CCL2+DPP4+ mesenchymal stem cells.

Mao et al. revealed a novel mechanism in which intestinal muscle layer cells are involved in the formation of CF. Their research indicates that muscle layer cells in patients with CD produce a unique extracellular matrix (ECM) scaffold. This scaffold maintains direct spatial and functional relationships with the overlying CF. Within this matrix, fibronectin has been identified as a key factor driving the migration of preadipocytes out of the mesenteric adipose tissue, thereby contributing to CF formation (63). In addition, a subpopulation of CCL2+DPP4+ mesenchymal stem cells (MSC1-S1) is significantly expanded within CF. Under the stimulation of CCL20+CD14+ monocytes and IL-6, this cell subset will accelerate its differentiation into dystrophic adipocytes, driving the abnormal formation of CF. The MSC1-S1 subgroup shows effective characteristics of promoting adipogenesis and fibrosis. Its metabolic activities involving sphingolipids, arginine, and nicotinamide are significantly upregulated. These features are positively correlated with active progression and poor prognosis in CD (**Figure 3**) (64).

### 5.3 CF, intestinal inflammation, and fibrosis

CF is not only closely associated with intestinal inflammation but also drives the fibrotic process through multiple mechanisms (65). The CF-derived microbiota such as *Klebsiella variicola* can exacerbate colitis by activating the type VI secretion system (66). CF secretes adipokines and various pro-inflammatory cytokines such as TNF-α, IL-1β, and IL-6, actively participating in the pathogenesis of intestinal inflammation. Compared to normal tissue, CF shows a significant increase in both immune and non-immune cells, particularly Th1, Th17, Treg, CD8+ central memory T cells, and M2 macrophages. Innate lymphoid cells (ILCs) within CF are the innate counterparts to CD4+ T cells. ILC2s regulate the recruitment of eosinophils and M2 macrophages within the adipose tissue. Leptin participates in the inflammatory and fibrotic processes by regulating the differentiation, function and metabolism of various immune cells and intestinal epithelial cells. At the same time, other adipokines are involved in immune regulation. These adipokines include ghrelin, resistin and visfatin (14). The intricate immune microenvironment within the adipose tissue and its interaction with intestinal inflammation jointly contribute to the progression of CD.

**Figure 4:**
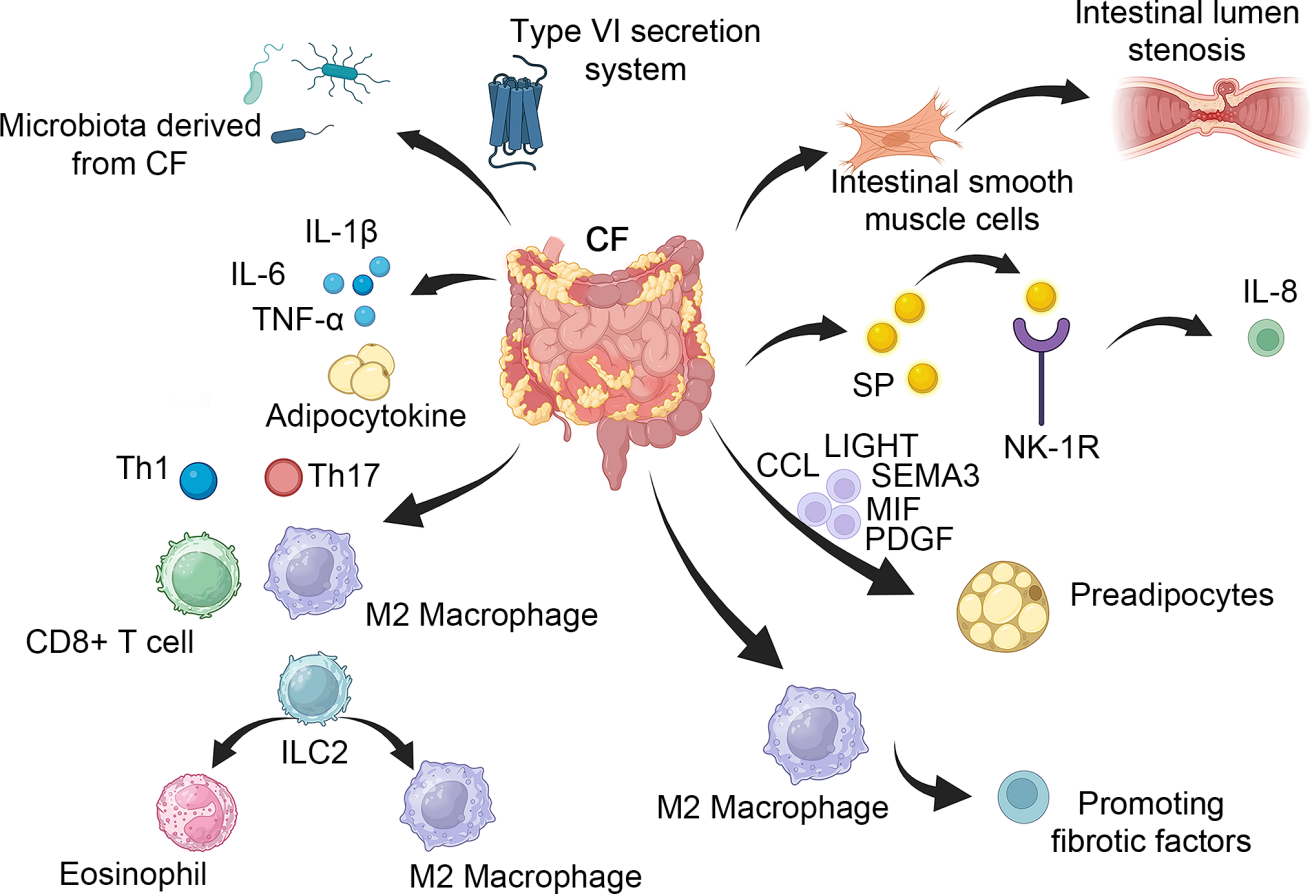
The role of CF in intestinal inflammation and fibrosis in CD. The CF-derived microbiota can exacerbate colitis by activating the type VI secretion system. CF secretes adipokines and various pro-inflammatory cytokines such as TNF-α, IL-1β, and IL-6, promoting intestinal inflammation. The Th1, Th17, Treg, CD8+ T cells and M2 macrophages in CF significantly increased. ILC2s in CF can regulate the recruitment of eosinophils and M2 macrophages in adipose tissue, jointly contribute to intestinal inflammation. CF promotes the proliferation of intestinal smooth muscle cells, thereby stimulating the hypertrophy of the muscular layer and causing the narrowing of the lumen. The expression of SP, NK-1R, and various pro-inflammatory cytokines is upregulated within CF. When SP binds to the NK-1R on the anterior mesenteric adipocytes, it will trigger the release IL-8, intensifying the inflammatory response. The intercellular communication mediated by CCL, LIGHT, MIF, SEMA3 and PDGF was significantly enhanced, while the PAC lineage aggravated intestinal inflammation. Fibrosis in CF is closely associated with the preferential polarization of M2 macrophages and their secretion of pro-fibrotic factors. Created with BioRender.com. CF, creeping fat; CD, Crohn’s disease; ILC2, Innate lymphoid cell 2; SP, Substance P; NK-1R, neurokinin receptor 1; PAC, preadipocyte.

CF is intricately linked to the pathogenesis of intestinal fibrosis and stricture formation. Sheehan et al. observed that fat wrapping was present in the majority of ileal and colonic resection specimens. The presence of fat wrapping is correlated with connective tissue alterations, including transmural inflammation, fibrosis, and stricture formation (67). Research by Bauer-Rowe et al. further confirmed that CF is a critical reservoir for pro-fibrotic fibroblasts, significantly propelling the progression of intestinal fibrosis in patients with CD (68). In addition, CF selectively promotes the proliferation of intestinal smooth muscle cells through the release of long-chain free fatty acids (FFAs). This leads to hyperplasia of the muscularis propria, which constitutes the structural basis for luminal strictures in CD (69, 70). What’s more, CF participates in CD-associated inflammation via the Substance P (SP)-neurokinin receptor 1 (NK-1R) signaling pathway. The expression of SP, NK-1R, and various pro-inflammatory cytokines is upregulated within CF. When SP binds to the NK-1R on the anterior mesenteric adipocytes, it will trigger the vigorous release of inflammatory mediators such as IL-8, thereby intensifying the inflammatory response (71). Hwang et al. further revealed the pro-inflammatory and pro-fibrotic roles of the preadipocyte (PAC) lineage within CF. Research demonstrates that the PAC lineage in CF exacerbates intestinal inflammation by highly expressing cytokines and responding to bacterial stimuli. At the same time, intercellular communication mediated by inflammatory and fibrosis-associated cytokines, such as CCL, LIGHT, SEMA3, MIF and PDGF, is also significantly enhanced (61). Additional studies have observed that fibrosis in CF is closely associated with the preferential polarization of M2 macrophages and their secretion of pro-fibrotic factors (**Figure 4**) (14, 58).

### 5.4 Application of CF in CD disease monitoring

The extent of CF proliferation is positively correlated with the severity of CD (72, 73). In recent years, the application of non-invasive imaging methods in the detection of CF has attracted widespread attention. Computed tomography enterography (CTE) serves as a potential non-invasive tool for assessing CF (74). Wei et al. indicated that the presence of CF is an independent predictor of poor response to infliximab treatment (75). Barajas et al. used MRE and the Magnetic Resonance Activity Index (MaRIA) to divide the patients into mild-to-moderate and severe disease groups. They found that CF is an independent risk factor for severe disease (OR = 11.50, p ≤ 0.001), suggesting its significant value in assessing disease severity (76).

Du et al. developed a surgical risk prediction model based on CTE images by extracting radiomics features from both the bowel wall and CF. The results showed that the AUC values of the CF model in the training and validation cohorts reached 0.916 and 0.822, respectively, indicating excellent discrimination ability. This model provided effective supplementary information to the bowel wall model. The combined model significantly improved predictive performance and could accurately predict the risk of surgery within one year following CTE in patients with CD (77).

The proliferation process of CF is often accompanied by active angiogenesis, which macroscopically manifests as an increase in new blood vessels. On CTE imaging, this phenomenon can be assessed indirectly through the characteristic comb sign. Li et al. proposed a CT-based Mesenteric Creeping Fat Index (MCFI). This index can perform semi-quantitative grading of CF by evaluating the degree of adipose tissue in the mesentery and the degree of vascular wrapping around the intestinal loops. Research indicates that the MCFI is highly correlated with the actual degree of fat wrapping observed in surgical specimens (r = 0.840, p = 0.000) (78). Zhou et al. confirmed that the MCFI is an effective indicator for predicting early postoperative recurrence in patients with CD. It demonstrates an AUC of 0.838, a specificity of 89.6%, a positive predictive value of 90.7%, and an accuracy of 83.6%. Furthermore, it was identified as an independent predictor in multivariate analysis (OR = 25.71, p < 0.001) (79). Studies have reported that higher MCFI scores are significantly associated with a reduced incidence of transmural healing. This establishes the MCFI score as a critical risk predictor for this outcome (80). Meng et al. developed a nomogram model by integrating the MCFI, mesenteric edema, and disease duration. This model showed greatest discrimination and the highest net clinical benefit in the test cohort. Based on these results, the model can be a reliable tool for assessing the severity of fibrotic strictures in patients with CD (81). In conclusion, these studies demonstrate that MCFI based on CTE can be an important tool for clinical follow-up.

Dual-energy CT enterography (DECTE) has emerged as a new non-invasive tool assessing CF through quantitative spectral parameters. Significant differences exist in the spectral parameters between CF and normal MAT. Due to the presence of edema and congestion, active CF is manifested on imaging as an increase in water content and iodine contrast agent concentration. As a result, relevant parameters such as λ_HU_, NFIC, NFWC, and NFVF show statistically significant differences between active and inactive phases, enabling an effective distinction of the disease’s active state (82). IUS enables the clear visualization of CF surrounding inflamed bowel segments in CD. On ultrasound images, this structure appears as a hyperechoic zone adjacent to the bowel wall. It typically originates at the mesenteric border and gradually encases the bowel wall as the disease progresses, serving as a critical imaging feature reflecting intestinal inflammatory activity. Zhou et al. reported that intestinal ultrasound and CTE have a high degree of consistency in detecting adipose tissue in the intestines, with a total accuracy rate of 88.2%. In patients with extensive lesion involvement, this rate increases to 93.4%. These findings indicate that IUS is a reliable and radiation-free diagnostic method for CF, especially suitable for assessing patients with active ileal or ileocolonic CD (74).

## 6 Research progress on blood lipids and CF in CD

Currently, the interaction between blood lipids and CF has not been fully elucidated. As a highly activated adipose tissue, CF is not only composed of various adipose lineage cells but also exhibits significant secretory functions, capable of releasing fatty acids and adipokines, suggesting its active lipid metabolism characteristics. Moreover, its formation mainly relies on adipocyte proliferation rather than hypertrophy, and this structural feature is typically associated with higher lipid metabolic activity and metabolic reprogramming. Combined with its spatial co-localization with the inflamed intestinal segment and the infiltration of immune cells, it indicates that CF may participate in immune regulation and tissue remodeling processes through lipid-mediated signaling pathways, demonstrating typical immune-metabolism coupling characteristics (83).

The stromal cell population in CF significantly upregulates the LPL and peroxisome proliferator-activated receptor (PPAR)-related pathways, suggesting enhanced lipid uptake and metabolic regulation capabilities. As key lipid-sensing nuclear receptors, PPARs can respond to fatty acids and their metabolites accumulated in the inflammatory microenvironment. By promoting fatty acid β-oxidation and inhibiting glycolysis, they reshape the metabolic pattern of immune cells, thereby driving macrophages to transform into anti-inflammatory M2 phenotype and promoting T cells to differentiate towards regulatory direction. Additionally, the PPAR signal further limits the inflammatory response by inhibiting pro-inflammatory transcriptional pathways. Functional knockout studies also indicate that the loss of PPAR-mediated metabolic balance will significantly amplify the inflammatory response mediated by Th1/Th17. These findings suggest that lipid metabolism in CF is not only a regulatory mechanism for local energy supply but also plays a regulatory role in the occurrence and development of Crohn’s disease through PPAR-mediated immune metabolic coupling (84).

Based on this, chronic inflammation can further drive the deep lipid metabolic reprogramming of CF, manifested as the coexistence of abnormal fat generation and lipid metabolic dysfunction. On one hand, the number of new adipocytes mediated by mesenchymal stem cells increases; on the other hand, the expression of lipid synthesis-related enzymes in these adipocytes decreases, resulting in impaired overall lipid storage capacity. At the same time, the downregulation of fatty acid dehydrogenase 2 disrupts polyunsaturated fatty acid metabolism and promotes the generation of pro-inflammatory lipid mediators. It is worth noting that lysophosphatidylcholine, as a key lipid metabolic mediator, accumulates significantly in CF and drives abnormal fat generation through the activation of the EGR2–PPARγ pathway, while enhancing oxidative stress and inflammatory responses. Moreover, it also weakens the immune function of adipocytes by inhibiting the expression of antimicrobial peptides, thereby exacerbating the persistence of inflammation and tissue fibrosis. These results further indicate that the lipid metabolic abnormalities in CF are not only manifestations of metabolic disorders but also participate in the occurrence and progression of Crohn’s disease through the immune-metabolic coupling mechanism (85).

Research indicates that the expression of lipolysis-related genes is specifically upregulated in MAT. This upregulation correlates with elevated levels of circulating FFAs, highlighting the pivotal role of MAT in regulating systemic lipid dynamics (86). As an important component of visceral adipose tissue, MAT exerts potent endocrine and paracrine functions. It is resistant to the lipolytic effect of insulin, leading to the release of FFAs and TGs into the circulation. This active lipolysis, combined with the impaired lipid buffering capacity after meals, collectively triggers the leakage of lipids from several insulin-sensitive peripheral tissues, including skeletal muscle, the liver, the pancreas, the heart, and the kidneys (87, 88). Wang et al. also discovered that mesenteric fat can directly release FFA into the liver through the portal vein, and significant reduction in mesenteric fat is often accompanied by decreased TG synthesis and liver lipid accumulation (89). Furthermore, due to the presence of CF, ileocolonic malabsorption may account for the reduction in blood lipids such as cholesterol (15).

At present, there are still relatively few studies on the relationship between lipid levels and the combined use of CF for evaluating the activity of CD. In general, CF establishes a crucial connection between the local inflammatory microenvironment and systemic lipid homeostasis through lipid metabolism reprogramming. Although the specific mechanism between CF and lipids still needs to be further clarified, the combined analysis of these two factors has potential application value in the assessment of CD activity. Currently, the related research is still limited. In the future, efforts should be focused on exploring the feasibility of integrating lipid indicators and CF characteristics for disease monitoring, and deeply analyzing the synergistic mechanism in the disease progression.

## 7 Discussion

The characteristic dyslipidemia observed during the active phase of CD is not merely a passive consequence of the disease. Rather, it is a manifestation of intimate interaction with the systemic inflammatory state (90). This metabolic change reveals a bidirectional regulatory relationship between immune inflammatory response and lipid metabolism, indicating that lipid metabolism not only reflects the disease state but may also directly participate in disease progression by regulating immune function and metabolic reprogramming. Based on this dual role, lipid metabolism is expected to serve simultaneously as a marker of inflammatory activity and a predictor of treatment response. However, current research on lipid metabolism in CD still has some limitations. Firstly, previous studies were mostly single-center and small-sample studies, with a relatively limited source of the research population. This not only restricted the generalizability of the results but also increased the risk of selection bias. Secondly, the standards for disease staging, treatment status, and control of comorbidities vary among different studies, which leads to a significant increase in heterogeneity. This consequently reduces both the reliability of research conclusions and the overall value of integrated analysis. Furthermore, most studies have adopted cross-sectional or retrospective designs, making it difficult to dynamically reflect the changes of blood lipids during the occurrence and development of the disease. It limits the inference of causal relationships and failing to clarify whether dyslipidemia is a result of inflammation or a key factor in disease progression. Additionally, existing studies lack standardized approaches for the selection and analysis of lipid indicators, with a focus on traditional indicators such as TC, LDL-C, and HDL-C. The limited attention given to more biologically significant indicators such as apolipoproteins, oxidized lipids, and lipidomic characteristics may underestimate the complex regulatory role of lipid metabolism in CD. In addition, the influence of potential confounding factors on research results has not been adequately controlled. For instance, nutritional status, intestinal absorption function, drug intervention, and liver metabolic function can significantly affect blood lipid levels, but they have not been adequately taken into account in some studies. Failure to conduct systematic corrections could lead to biased or erroneous research results. Furthermore, differences between various detection methods and experimental platforms may introduce measurement bias, further affecting the stability and reproducibility of results. In terms of the mechanism, the functional differentiation of different lipid subtypes in the intestinal inflammatory microenvironment remains unclear. In particular, the specific mechanisms of action in immune cell polarization, amplification of inflammatory signals, and intestinal barrier damage still need to be further explored.

At the same time, as a unique pathological feature of CD, CF is far more than a simple mechanical wrapping. It drives the progression of intestinal inflammation, fibrosis, and stricturing complications through complex interactions with the microbiome, the immune system, and mesenchymal cells (14, 66). Therefore, CF is not only a structural indicator of disease progression, but is also likely to be an important driving factor for the persistence of inflammation and tissue remodeling. With the development of modern imaging technologies such as CTE, MRE and IUS, it has become possible to conduct non-invasive and quantitative assessment of CF, facilitating its transformation from qualitative descriptive indicators to quantifiable and traceable clinical parameters. Therefore, the MCFI and radiomics models developed based on these technologies have demonstrated good predictive value for disease severity, surgical risk, and postoperative recurrence (79, 91, 92). However, these methods still encounter numerous challenges in clinical routine applications. For instance, the insufficient repeatability of radiomics features limits the comparability of the results. At the same time, the reliance on specialized software and technical personnel increases the threshold for practical application. Differences in image quality and the lack of a standardized quantitative evaluation system lead to fluctuations in relevant indicators across different studies. Moreover, the cost-effectiveness and clinical application value of integrating these technologies into routine diagnosis and treatment pathways have not been fully verified, thus restricting their widespread application in routine diagnosis and treatment.

Based on these deficiencies, future research needs to achieve breakthroughs at multiple levels. Firstly, at the basic research level, a systematic analysis of the molecular functions of different lipid components in the intestinal inflammatory microenvironment should be conducted, with a focus on their key role nodes in immune regulation, inflammatory signal transduction, and fibrosis processes. Secondly, at the mechanism research level, the causal relationship between abnormal lipolysis of mesenteric fat and systemic lipid metabolism disorders needs to be further clarified. At the clinical research level, multi-center, large-sample, and prospective cohort studies will be needed to verify the stability and generalization ability of the combined application of lipid indicators and CF in disease assessment and prognosis prediction. On this basis, a multi-parameter prediction model integrating lipid indicators, CF imaging features, and other biomarkers will provide strong support for the individualized and precise management of CD. The ultimate goal is to establish a dynamic monitoring system centered on non-invasive imaging and serum markers, thereby gradually reducing the reliance on invasive examinations and improving the long-term management and quality of life of CD patients.

## 8 Conclusions

In conclusion, CD has a high risk of disability and long-term burden, so disease monitoring is extremely important. Currently, the methods used to assess disease activity mainly include endoscopy, imaging examinations, and biomarkers. These methods all have certain limitations, such as invasiveness, high cost, or inability to comprehensively reflect intestinal wall and mesenteric lesions. More and more evidence shows that lipid metabolism abnormalities and CF play a key role in the pathogenesis, progression, and prognosis assessment of CD. Abnormal lipid levels during the active stage not only reflect systemic inflammation but also indicate intestinal metabolic disorders. CF actively participates in the formation of intestinal inflammation, fibrosis, and intestinal stenosis through immunological, microbial, and molecular mechanisms. The development of imaging techniques such as CTE, MRE, and IUS has made semi-quantitative assessment of CF possible and applicable for predicting surgical risks and postoperative recurrence. The combined application of lipid levels and CF assessment provides potential non-invasive methods for CD activity monitoring, disease severity assessment, and prognosis prediction. In the future, it is necessary to further clarify the interaction mechanism between lipid metabolism and CF and verify the combined model based on lipid and CF in large-scale clinical cohorts to provide individualized, dynamic, and precise strategies for CD management, thereby improving patient outcomes and long-term prognosis.
